# A within-study cross-validation of the values-as-ideals measure: levels of value orientation explain variability in well-being

**DOI:** 10.1016/j.heliyon.2022.e12131

**Published:** 2022-12-09

**Authors:** Anastasia Besika

**Affiliations:** Department of Psychology, University of Zurich, Zurich, Switzerland

**Keywords:** Values, Levels of value orientation, Well-being, Latent profile analysis, Meaning in life

## Abstract

There is a consensus that values serve as ideal standards that motivate and influence behavior. Previous research concludes that certain universal values promote well-being and others undermine it. In line with the idea that values behave as a dynamic system and do not influence well-being as independent elements, the present findings indicate that all universal values may contribute to well-being. A new measure assessing the degree 10 universal value domains serve as ideals is administered on an online sample (*N* = 933) from the United Kingdom. Participants completed three well-being measures. Latent Profile Analysis in a within study cross-validation (Sample 1: *n* = 468, Sample 2: *n* = 465) replicates three distinct latent value profiles denoting *high*, *moderate* and *low* levels of value orientation. Analysis of Variance shows that the level of value orientation explains differences in average levels of well-being. A *high*-level of value orientation is associated with higher average levels of well-being compared to a *low*-level of value orientation. This evidence suggests that the degree values influence well-being depends on the level they represent people's ideals. In conclusion, the type of value pattern and not the type of prioritized values can systematically explain variability in well-being. Implications are discussed.

## Highlights

• Providing a new methodology for assessing the relationship between values and well-being.
• There are distinct latent value profiles that denote three levels of value orientation.
• Levels of value orientation explain differences in average levels of well-being.
• A high level of value orientation is healthier that a low level of value orientation.
• Values influence well-being as a dynamic pattern not as independent elements.

## 1 Introduction

**Figure 1:**
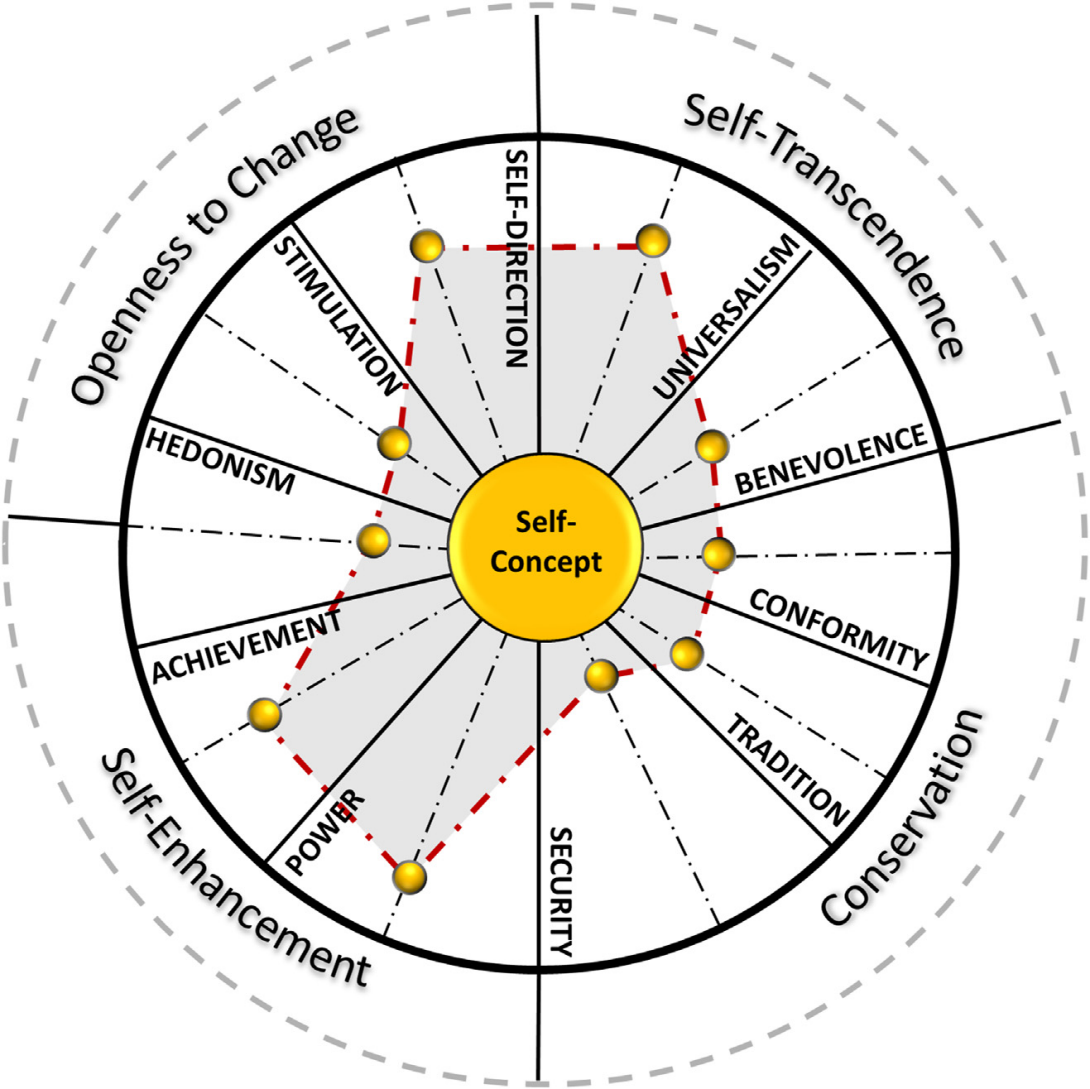
Individual value pattern organized within the universal structure of values in relation to self-concept.

Values constitute universal meaning systems (Lempp, 2014) and serve as ideals that inform personal goals (Castro et al., 2016; Pitt, 2014). Findings suggest that people share a circular structure of values, within which they organize their value priorities according to how close each value is to their self-concept (Besika et al., 2021; Cieciuch et al., 2016; Schwartz, 1992; Verplanken and Holland, 2002) (Figure 1). People re-adjust the organization of their value priorities systematically in response to change (Bardi, 2009). However, previous studies that investigate the relationship between values and well-being focus only on the values people report as mostly important at the time of measurement (e.g., Kasser and Ahuvia, 2002). Despite this line of investigation reporting inconsistent results across studies, regarding the size and direction of the associations between single values and well-being measures (Schwartz and Rubel, 2005; Sortheix and Lönnqvist, 2013; Sortheix and Schwartz, 2017; Verkasalo et al., 2009), researchers infer that certain values are “healthy” whereas others are “unhealthy”. As negative associations between values and well-being measurements are not replicated across studies (e.g., Bobowik et al., 2011), the present work tests the overarching hypothesis that values influence well-being as a pattern and not as single elements and aims to identify the kind of value pattern that is associated with high levels of well-being. The present article introduces a new methodology for assessing the relationship between values and well-being. Two studies replicate three distinct value patterns, interpreted to denote *levels of value orientation* (LVO). Analysis reveals significant differences in the average levels of well-being across LVO groups, and high levels of value orientation are associated with high levels of well-being. In line with recent explorative studies (Besika et al., 2022), the present findings provide evidence that values influence well-being as a dynamic pattern. The new methodology for investigating the relationship between values and well-being dissolves previous conceptual dichotomies of the circular structure of values (Schwartz, 1992). The present findings support the idea that even though value priorities may be pertaining to predicting personal goals and behavior (Sagiv and Roccas, 2021), values do not behave as independent elements as they constitute a universal cognitive system.

Over the last 30 years, intercultural studies indicate a universal circular structure within which people organize their value priorities (Cieciuch et al., 2016; Schwartz, 1992). Researchers assume that the circular continuum denotes the relationship between values, where adjacent values are complimentary and those in opposite positions within the circular structure are conflicting. This conceptual division derives from the assumption of an inherent value conflict where a personal (self) interest underlies certain values and a social (others) interest underlies other values (Schwartz, 1992). Nevertheless, further studies indicate that the *self* and *others* dimensions may constitute a separate latent pattern that mediates the value-behavior relationship (Besika et al., 2021; Gaertner et al., 2008). Moreover, longitudinal studies (e.g., Bardi et al., 2009) report that changes in value priorities facilitate adaptation on a macro-time frame (Freund and Ritter, 2014). Thus, as value priorities fluctuate at a within-person level across time, certain values may display positive associations and others may display negative associations with well-being at a given point (Besika et al., 2022). Adopting a system dynamics perspective and drawing on the above, this article suggests that it is the overall level the universal values serve as a person's ideals that provides meaningful information regarding their well-being and not the values they prioritize. Altogether, this work suggests that: (1) The dichotomy of the universal structure of values into “healthy” and “unhealthy” is based on inconsistent findings. (2) Several factors may mediate the value to well-being relationship. (3) Values behave as a pattern within which single values complement each other in their underlying function to produce goal-directed behavior. (4) All universal values may contribute to well-being.

### 1.1 Values as a dynamic system

Humans acquire values throughout their development and socialization (e.g., education, parenting, occupation, etc.) and thus, values are not innate characteristics (Hofstede, 1980). Through ongoing individual and shared experiences and processes that do not typically involve a conscious engagement, people internalize values that represent their socio-cultural content and context (Maio, 2010; Maio et al., 2009; Rohan, 2000; Schwartz, 2016). Research indicates that regardless of culture, people assign similar meaning to 57 values that are structured within the domains of Conformity, Tradition, Security, Power, Achievement, Hedonism, Stimulation, Self-direction, Universalism and Benevolence (Schwartz, 1992, 2006). Values are interrelated and behave as a system that fluctuates across the life span to facilitate adaptation (Allicock, 2008; Bardi et al., 2009; Freund and Ritter, 2014).

### 1.2 Conflicting values

Researchers interpret the systematic behavior values display to indicate either a complimentary or a conflicting relationship amongst certain values. Hence, they consider adjacent values (e.g., Achievement and Hedonism) as “complimentary” and values occupying opposite positions in the circular continuum (e.g., Benevolence and Achievement) as “conflicting” (Schwartz, 1992). Accordingly, researchers dichotomize the circular structure of values based on the distinct dimensions of *individualism* and *collectivism* (Johnston, 1995), assuming that values have either a *personal focus* (e.g., Achievement) or a *social focus* (e.g., Benevolence). However, the interpretation of the circular continuum as a representation of compatibilities and conflicts amongst values constitutes a conceptual discrepancy. Having conflicting parts within a dynamic system is not in line with the functionality principles of dynamic systems. Since all parts of a dynamic system interact in aiming to produce behavior (Ford, 1999) all values are complimentary.

Furthermore, such a dichotomy cannot be applied to a circular structure as it leaves unexplained the relationships between adjacent domains, which form the ends of the two semi-circles. For example, if adjacent values are complimentary and opposite values conflicting, what is the relationship between Self-Direction (self-focus) and Universalism (social-focus) and between Power (self-focus) and Security (social-focus), as they are adjacent and at the same time fall within the two divisions? Schwartz (1992) suggests that Security is an exception containing mixed motivations as it concerns both personal and social safety, harmony and stability. This implies that Security can motivate people to serve both their own interest and the interest of others. Is there a valid reason that prevents all values from having a dual motivation? For example, could not Conformity equally motivate a person to strive for being obedient, polite and honoring others as well as for successfully integrating in their social environment? Evidence indicates that a single value may incorporate both motivations. For example, the value of altruism (i.e., having an unselfish desire to enhance the welfare of others) within the domain of Universalism, incorporates the motivation to serve the interest of others as well as personal interest, since the end-goal of an altruistic action can generate personal pleasure and lead to actions that serve others (Batson and Shaw, 2009; Hubbard et al., 2016). Similarly, any value may have the potential to motivate a person toward either or both orientations.

### 1.3 Healthy vs unhealthy values

A further division of values into “healthy” vs “unhealthy” derives from the Self-Determination Theory (STD; Ryan and Deci, 2001) and considers certain values to promote and others to undermine well-being (Kasser and Ahuvia, 2002; Kasser and Ryan, 1996; Schwartz, 2016; Sortheix and Schwartz, 2017). According to SDT, satisfying the three intrinsic needs (i.e., needs deriving from within the person) of *autonomy*, *competence* and *relatednes*s leads to self-actualization (i.e., reaching optimal health and thriving). The value domain of Self-Direction pertains to *autonomy*, the value domain of Achievement to *competence*, and both the value domains of Benevolence and Universalism pertain to *relatedness* (e.g., Sagiv and Schwartz, 2000). The values domains of Security, Conformity, Power and Tradition are considered “unhealthy” as they are related to extrinsic needs (i.e., driven by external rewards). Studies investigating the relationship between values and well-being report a positive association of subjective well-being measures with certain values (i.e., Self-Direction) and a negative association with other values (e.g., Conformity). For example, financial success is associated with low social productivity and behavioral disorders (Kasser et al., 2014). Following an STD perspective, researchers conclude that pursuing “healthy” values may increase well-being, whereas pursuing “unhealthy” values may decrease positive affect and life satisfaction (Schwartz and Sortheix, 2018). In spite of results not being replicated, researchers divide the value circular continuum into *Growth Self-Expansion Anxiety-Free* (healthy) and *Self-Protection Anxiety-Control* (unhealthy) values.

However, characterizing certain universal values as “unhealthy” generates further theoretical discrepancies. Firstly, it undermines theoretical assumptions that underpin Schwartz's model, who states that “values are beliefs linked inextricably to affect. When values are activated, they become infused with feeling. People who value independence highly are aroused if their independence is threatened, feel despair when they are helpless to protect it, and are happy when they can exercise or use it” (Schwartz, 2010, pp. 222–223). Studies show that the same reasoning applies to people who value health, which is within the domain of Security. Experimental research shows that people who value health highly are aroused if their health is threatened, feel despair when they are unable to protect it, and are happy when they are healthy, as it allows them to enjoy many other life domains (Allicock, 2008). Secondly, the idea of “unhealthy” values contradicts previous findings in line with theories such as the Cognitive Dissonance Theory (Festinger, 1957) and the Self-Discrepancy Theory (Higgins, 1987). For example, a piece of evidence shows that pursuing goals that are congruent with personal values is associated with positive affect, life satisfaction and meaning (McGregor and Little, 1998). Additional findings suggest that satisfaction is positively associated with the degree a person is successful in the domain they value most, regardless of its associated value (Oishi et al., 1999).

Could the sporadic negative associations between certain values and well-being measures be due to other aspects of the overall psychological functioning and not due to inherent characteristics of values? As values represent a dynamic system (Rokeach, 1973) changes in life tasks influence the nature of what people consider to be important (Erikson, 1963; Freund and Ritter, 2014). A particular value may not serve an important life task at a given point in time. However, it may become important at another point in time. For example, *John* was happy to leave his family home to pursue an academic career in another continent, as he used to value Achievement more than Benevolence. When *John* started his own family and became a father, he prioritized Benevolence and decided to settle in a position that did not promise him a very rewarding career, research wise, however it provided security and stability for bringing up his child and growing his own family. Thus, when a value forms part of a person's value system it may influence their behavior to a lesser degree than a value that has a higher level of importance. However, a situation can activate a less prioritized value and change its priority within a person's system. As value priorities fluctuate across the life span (Bardi et al., 2009; Freund and Ritter, 2014) it is possible that all universal values that serve as guides in peoples' lives, regardless of cultural context (Schwartz and Cieciuch, 2021) may have a protagonist role at different time points in a person's life.

### 1.4 Values and well-being

Research findings show that actions taken in the simultaneous pursuit of certain values are compatible when pursuing some values and conflicting when pursuing others. For example, actions that express conformity may compliment actions that express security, and they may conflict with actions that strive for independence (Schwartz, 1992). Does this imply an inherent conflict between the values of Security and Independence? Assuming that values represent what is meaningful to people, with each value representing different meanings (Reker and Wong, 1988), and considering that values influence well-being as patterns (Besika et al., 2022) may lead to alternative interpretations of the systematic behavior observed in values. Accordingly, the negative association of certain values (e.g., Power and Achievement) to well-being measurements that are randomly observed (e.g., Sagiv and Schwartz, 2000) may be due to other factors influencing this relationship. In the following, some factors are discussed indicatively as possible mediators/moderators in the relationship amongst values as well as in the values to well-being relationship: a) value extremity, b) value congruence, and c) a range of psychological factors.

### 1.5 Value extremity

Extreme endorsement of a value may influence the levels of psychological tension people experience. For example, valuing Freedom too much may lead to anarchy; high centrality of Achievement may lead to workaholism. *Sacred values* (i.e., values of high importance and with infinite significance) do not allow for any trade-offs (Tetlock, 2003). When people are forced to compromise their sacred values*,* they become psychologically disturbed. An internal moral obligation to protect a sacred value leads to neglect of the consequences of an action. Even the thought of a sacred value's violation can lead people to experiencing moral outrage, anger, contempt and extreme behavior (Tetlock et al., 2000). Generally, extremes are associated with poor social relationships, exhaustion and harmful behaviors (e.g. suicide bombers) (Killinger, 2006; Schaufeli et al., 2008).

### 1.6 Value congruence

Value congruence between individuals and their environment moderates the value-life satisfaction relationship (Sagiv and Schwartz, 2000; Sortheix and Lönnqvist, 2013). Studies show that values categorized as “unhealthy” (e.g., Power), may be positively associated with life satisfaction when they are endorsed by people's social environment (e.g., Musiol and Boehnke, 2013). In addition, studies indicate that activities associated with Conformity (categorized as “unhealthy”) may serve as sources of satisfaction to people who value Conformity (Oishi et al., 1999). Overall, pursuing value-congruent goals is positively associated with well-being, regardless of the value type (McGregor and Little, 1998).

### 1.7 Psychological factors

#### 1.7.1 Unfulfilled needs

People change their value priorities in response to their life circumstances and their developmental stage (Verhaeghen et al., 2014). Unmet psychological needs (e.g., low self-esteem) may motivate a person to pursue success in seeking external validation and not values of Achievement *per se* (Vyskocilova et al., 2015). Similarly, reward expectations may motivate benevolent acts (i.e., warm glow) (Andreoni, 1990) and not values of Benevolence *per se*. Longitudinal studies show that young children develop values of Security first, as they need to feel safe in their environment. At a later stage of development and as children start exploring the outside world, they develop further values such as Self-Direction (Cieciuch et al., 2016). As value priorities fluctuate in response to psychological needs and developing security precedes independence (Ainsworth, 1973; Bowlby, 1969; Schwartz, 1992), prioritizing the value of Security due to an unfulfilled need to feel safe, may undermine psychological development and well-being. A piece of evidence supports the idea that satisfaction of psychological needs mediates the relationship between values and life satisfaction (Kasser et al., 2014).

#### 1.7.2 Goal conflict

Adopting goals with incompatible strategies or unattainable end-states may give rise to goal conflict (Gorges and Grund, 2017). Conflict may also be the outcome of pursuing multiple goals, which are part of the same goal structure. A goal structure may consist of multiple sub-ordinate goals of competing nature and draw on limited resources (e.g., time, energy and money) even when they serve the same value (Kruglanski et al., 2002). Conflict may also arise between social roles. Despite their co-facilitating nature, roles in different life domains draw on limited time, energy and support resources (Frone, 2003).

#### 1.7.3 Priority fluctuation

As people value many things and they aim to satisfy everything that matters to them, values fluctuate on a continuum of *self*- and *others*-interest (Reiss, 2004). For example, a person may need to spend time alone after spending time in social gathering and having satiated their need to be with other people. This is in line with the view that focusing both on the *self* and on *others* may serve an individual, who is at the receiving end of all of their actions (Carver and Scheier, 2001). Perceived discrepancies between *self* and *others* generate emotional discomfort that leads to either cognitive or behavioral re-adjustments (Brandtstädter and Greve, 1994; Higgins, 1997).

#### 1.7.4 Social pressures

Cross-sectional findings indicate that the socio-economic and cultural contexts are additional factors that moderate the relationship between values and well-being (Sortheix and Schwartz, 2017). Social pressures may generate psychological conflict that is associated with certain value-related behaviors. For example, Western orientated cultures endorse individualism versus common good (Hofstede, 2001) and equate values such as success with acquisition of money, power and prestige (Brewer and Porter, 2013). Accordingly, these endorsements shape perceptions of happiness, which constitute of wealth, health and an anxiety-free life (Lu et al., 2001). People may struggle with the pressure of reaching the high standards of success and power set by their social environment. In addition, people who live in multicultural societies may experience psychological conflict due to receiving polarized messages within a globalized world.

### 1.8 Assessing the relationship between values and well-being

Measures such as the Schwartz Value Survey (Schwartz, 1992) and the Portrait Values Questionnaire (PVQ; Schwartz, 2006) serve to identify people's value priorities and to investigate their relationship to behavior. Researchers also employ these or similar measures to investigate the relationship between values and well-being (e.g., The Pairwise Comparison Value Survey; Oishi et al., 1998). Overall, the current measures entail ranking or rating scales that prompt respondents to indicate their value priorities in an indirect way. For example, the item in the PVQ measuring Achievement states: “It is important to him/her to show his/her abilities. He/she wants people to admire what he/she does”. Respondents answer the question: “How much like you is this person?” This indirect evaluation by comparison to an “ideal person” may tap into respondents' perception of how far they are from fulfilling that value, which may influence responses on well-being scales. Could questions that prompt direct identification with values as ideal generate different responses? In addition, Schwartz (2016) mentions that overall people seem to rate all values either high or low, which may imply that value priorities are not meaningful in isolation from their interrelated values. Could latent value profiles provide more meaningful and systematic information regarding the association between values and well-being than value priorities? A recent study makes a step toward this direction (Schmidt et al., 2021) by using Latent Profile Analysis (LPA) to identify clusters that characterize distinctive typologies based on responses on the PVQ. However, the scope of the study is to demonstrate that LPA can extract more than the recommended 3 to 5 number of profiles (Tein et al., 2013). Results of both Variational Bayesian LPA and Maximum Likelihood LPA identify *K* = 8 value profiles. As the authors characterize these profiles informed by the highest value mean within each cluster (e.g., “negative to all values but especially negative to power”), the eight profiles and their interpretations (e.g., strongly positive only to growth) do not provide additional meaningful insights further to Schwartz's (1992) four motivational orientations (e.g., openness-to-change).

### 1.9 The present work

Considering the dynamic nature of values from a system dynamics perspective (Ford, 1999) the present work assumes that an individual constructs a unique value pattern within the universal circular continuum that denotes the importance they assign to each value. On the one hand, this pattern helps a person align their self-perceptions, goals and actions to their social context (Maio, 2010). On the other hand, a person's value pattern allows them to adapt to situational or environmental change by re-adjusting their value priorities (Bardi et al., 2009). These assumptions inform the overarching hypothesis that all values within the universal structure may contribute to well-being. Hence, the present work goes beyond value priorities and introduces a new methodology for assessing the relationship of the 10 universal value domains to well-being. The main objective of the two studies is to identify people's value patterns and their relationship to well-being measurements. Latent Profile Analysis (LPA) using as indicators the 10 items of a new measure, which assesses *values-as-ideals*, identified three distinct latent value profiles that denote levels of value orientation. A within-study cross-validation tests the following three hypotheses.

• *Hypothesis 1:* Three distinct levels of value orientation (LVO) describe the data.
• *Hypothesis 2:* Average levels of well-being measurements distinguish the three LVOs.
• *Hypothesis 3:* There are significant differences in average levels of well-being measurements between participants in the *low*-LVO and *high*-LVOs.

## 2 Materials and methods

### 2.1 Participants and procedures

The sample size was determined on a 3-profile model, since previous work (Besika et al., 2022) indicates that three levels of value orientation may provide meaningful information regarding people's levels of well-being. Monte Carlo stimulation recommends a minimum sample size of 250–500 for a small (*d* = .2) to medium effect (*d* = .5), corresponding to the standardized distance between the means of 10 indicators (Tein et al., 2013). Priori power analysis using G∗ Power (Faul et al., 2007) showed that 400 participants would suffice for detecting a small to medium effect size *f* = .25, with high power .95 and α = .05 for three groups, when conducting ANOVA fixed effects, omnibus, two-way. Data collection adhered to ethical guidelines provided by the University of Zurich Ethics Committee of Human Subjects. Prior to entering the study, participants gave their informed consent. Participants who met the inclusive criteria of being a native English speaker without having previously reported mental health issues entered the online questionnaire by joining an online survey platform for a small monetary reward. Participants (Table 1) resided in the United Kingdom and their age ranged from 18 to 68 years with equal distribution of sex across three age groups of 18–34, 35–50 and 51–68. Splitting the data set into two subsets allowed cross-validation comparisons between Sample 1 (*n* = 468) and Sample 2 (*n* = 465). Analysis was conducted in R Core Team (2020). LPA with the package tidyLPA (Rosenberg et al., 2018) and poLCA (Linzer and Lewis, 2011) tested *Hypothesis 1*. Analysis of Variance (ANOVA) with the aov function (Chambers et al., 1992) tested *Hypothesis 2*. Multiple pairwise comparisons with the package multicomp (Gelman and Hill, 2006) tested *Hypothesis 3*.

**Table 1:** Sample descriptive statistics.

Sample	Participants	Age M	Age SD	Females
*N*	933	43.51	13.42	466
Sample 1	468	43.51	13.42	234
Sample 2	465	43.08	13.80	232

*Note. N* = total sample; Age M = age mean; Age SD = age standard deviation.

### 2.2 Measures

#### 2.2.1 Values-as-ideals

The new measure consists of 10 self-related statements (see Table 2 and Supplementary file) that assess the degree to which 10 universal value domains (Schwartz, 1992) serve as people's ideals. Describing each value with self-related statements aimed to activate participants' values, since those are not typically salient in people's awareness (Verplanken and Holland, 2002). Three independent professionals who were experts in well-being assessment and values validated the content of the statements and unanimously agreed on their appropriateness. In ensuring instrument clarity, 25 volunteer members of the public (age *M* = 45.22, *SD* = 11.56, females = 12) helped define the statements as common expressions that evoke intuitive responses. The statements described each value with simplicity (e.g., “In an ideal world, I feel safe wherever I am”; Security) (Sample 1, α = .77; Sample 2, α = .81) and all volunteers agreed that there were no ambiguities regarding the meaning of the items (Barker et al., 2015). In contrast to existing measures, participants rated intuitively the 10 statements without comparing their level of importance.

**Table 2:** Values-as-ideals measure.

No	Item	Value domain
1	I decide about which way my life goes.	Self-direction
2	I discover new things in life.	Stimulation
3	I enjoy life to the fullest.	Hedonism
4	I am successful in everything I do.	Achievement
5	I have resources and influence over others.	Power
6	I feel safe wherever I am.	Security
7	I respect other people and follow social rules.	Conformity
8	I accept and follow the ideas of my culture or religion.	Tradition
9	I care about my family, friends and others around me.	Benevolence
10	I care about all things on the planet	Universalism

*Note.* The descriptions of the 10 value domains are based on Schwartz's (1992) model of values and follow the stem item: *In an ideal world…*.

#### 2.2.2 Well-being

Three well-being measures served as the outcome variables of the study. (1) The Satisfaction with Life Scale (SWLS; Diener et al., 1985) (Sample 1: α = .92; Sample 2: α = .92) consists of five items measuring life satisfaction (e.g., “In most ways my life is close to ideal”). The scale has been used to investigate value associations to well-being (e.g., Schwartz and Sortheix, 2018). (2) The Multidimensional Existential Meaning Scale (MEMS; George and Park, 2017) involves 15 items that measure three facets of meaning in life: Comprehension (e.g., “My life makes sense”) (Sample 1: α = .94; Sample 2: α = .94), Purpose (e.g., “My direction is life is motivating”) (Sample 1: α = .91; Sample 2: α = .92), and Mattering (e.g., “I am certain that my life is of importance”) (Sample 1: α = .88; Sample 2: α = .88). The scale is associated positively with the SWLS (King et al., 2006; Steger and Kashdan, 2007). (3) The Perceived Stress Scale (PSS; Cohen et al., 1994) (Sample 1: α = .90; Sample 2: α = .89), that is negatively associated with the SWL (Atanes et al., 2015) consists of 10 items measuring perception of stress (e.g., “Felt unable to control the important things in life, over the past month”). Participants rated all measures on a scale from 1 = *not at all* to 7 = *very much* or 1 *=* or *strongly disagree* to 7 = *strongly agree*.

## 3 Results

### 3.1 Study 1

#### 3.1.1 Values-as-ideals reliability

The *values-as-ideals* measure displayed very good internal reliability (α = .77).

#### 3.1.2 Three distinct levels of value orientation (hypothesis 1)

**Figure 2:**
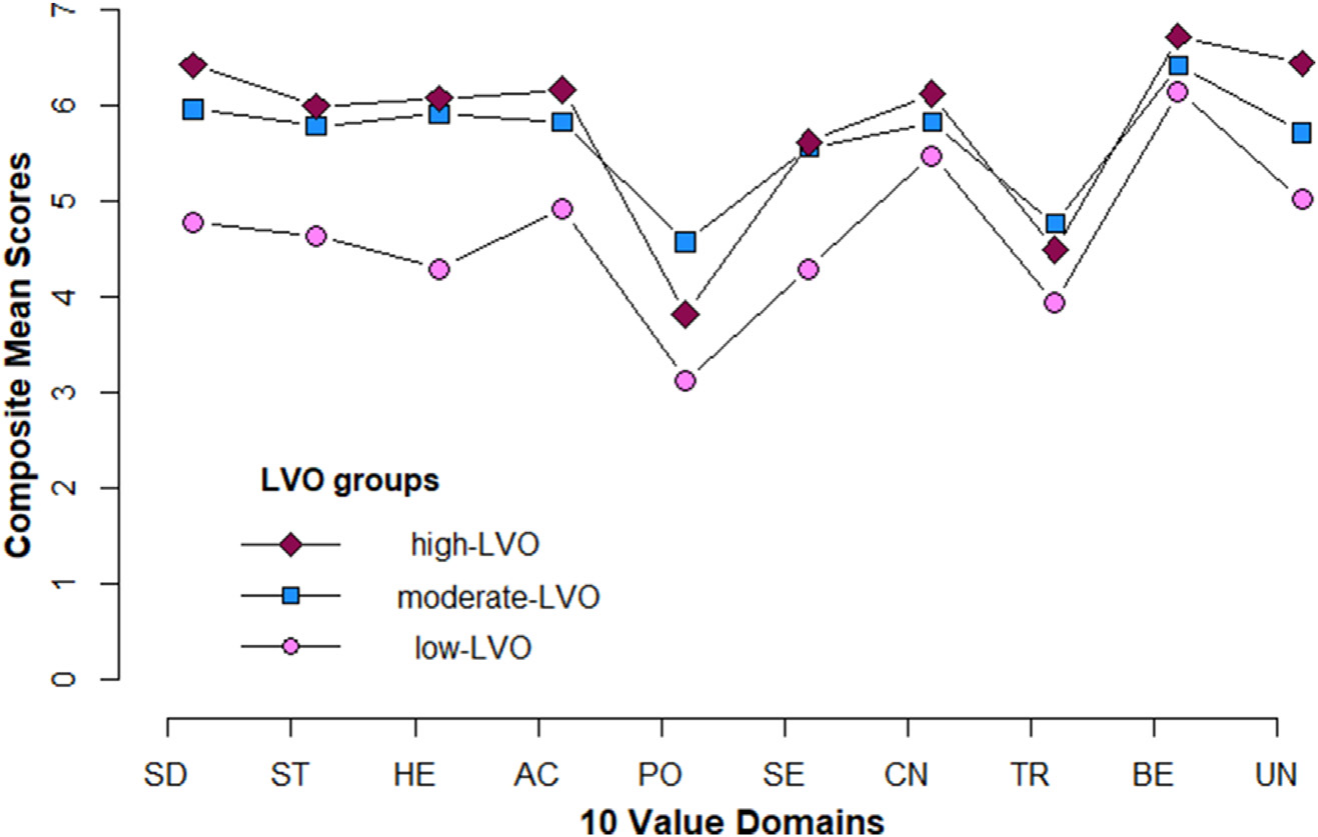
Sample 1 **|** Mean patterns of values across three groups distinguished by level of value orientation.

In Sample 1 (Table 1) *LPA* helped identify unobserved subgroups of participants that were differentiated based on systematically diverging rating patterns of the 10 *values-as-ideals* items. As it is common, the fit indices of 1- up until 5-profile models did not converge on one solution (Nylund-Gibson and Choi, 2018). Although, most fit indices continued to decrease down to a 5-profile model, indicating that an additional profile may result in a better solution, *Entropy* that informs how distinct the identified groups are, had the highest value in the 4-profile model (Oberski, 2016) (Table 3). However, the proportion of participants estimated to be in the smallest group of a 3-profile model was over 23% *(n.min* = .239), which made it more reliable than the 4-profile model *(n.min* = .124) (Nylund-Gibson and Choi, 2018; Tein et al., 2013). Thus, the 3-profile model was deemed to represent the data best. In the 3-profile model 35.80% of participants (*n* = 171, age *M* = 46.23, *SD* = 12.89, females = 89) were estimated to belong to profile 1; 40.64% of participants (*n* = 185, age *M* = 41.83, *SD* = 12.21, females = 88) were estimated to belong to profile 2; and 23.56% of participants (*n* = 112, age *M* = 43.93, *SD* = 14.06, females = 57) were estimated to belong to profile 3. The total percentage of item response probabilities across profiles indicated that on a 1 (*not at all*) to 7 (*very much*) point scale, profile 1 had its highest percentage of ratings between 4 and 5, profile 2 had its highest percentage of ratings between 5 and 6; and profile-3 had its highest percentage of ratings between 6 and 7 (Supplementary: Table 1). Informed by the structure of participants’ response patterns on the 10-item measure, a meaningful interpretation (Nylund-Gibson and Choi, 2018) was that the three latent profiles denote levels of value orientation. Accordingly, a *low-, moderate-* and a *high-level of value orientation* (LVO) described the data. Figure 2 shows that interestingly, the mean patterns of the 10 *value-as-ideals* items had a similar shape in all three profiles, with Power being the lower boundary and Benevolence the upper boundary.

**Table 3:** Study 1: fit statistics of latent profile analysis models as indicated by the *values-as-ideals* 10-item measure.

K	LL	BIC	SABIC	CAIC	AWE	Entropy	n_min
1	−6636	13394	13331	13414	13575	1	1
2	−6345	12881	12783	12912	13163	.75	.361
3	−6291	12839	12706	12881	13222	.76	.239
4	−6167	12660	12492	12713	13143	.85	.124
5	−6149	12692	12489	12756	13276	.76	.043

*Note. K* = number of profiles; *LL* = log-likelihood; *BIC* = bayesian information criterion; *SABIC* = sample size-adjusted BIC; *CAIC* = consistent Akaike information criterion. *AWE* = approximate weight of evidence; *Entropy* = a measure of classification uncertainty; *n*_min = proportion of the sample assigned to the smallest profile.

#### 3.1.3 Levels of value orientation distinguished by average levels of well-being (hypothesis 2)

Dummy coding LVOs into a three-level categorical variable allowed performing ANOVA and identifying differences in the average levels of well-being across the three groups. As indicated by the interclass and intraclass correlation coefficients (ICC1 and ICC2), the measurements of well-being were non-independent in LVO groups, which were distinguishable by their average levels of well-being variables (James et al., 1984; LeBreton and Senter, 2008) (Table 4).

**Table 4:** Study 1: ANOVA results and ICCs showing well-being measurements are non independent across LVOs.

Outcome	ICC(1)	ICC(2)	low-LVO		moderate-LVO		high-LVO		F(2, 468)	p
			M	SD	M	SD	M	SD		
Comprehension	.16	.97	3.98	1.33	4.91	1.28	5.20	1.28	30.57	<.001
Purpose	.20	.97	4.03	1.37	5.07	1.46	5.26	1.46	39.75	<.001
Mattering	.12	.95	3.36	1.41	4.22	1.46	4.53	1.46	22.28	<.001
SWLS	.17	.97	3.42	1.35	4.54	1.35	4.59	1.35	33.35	<.001
PPS	.02	.78	4.05	1.27	3.71	1.26	3.60	1.26	04.59	.010

*Note. LVO* = level of value orientation; *ICC(1)* = interclass correlation coefficient; *ICC(2)* = intraclass correlation coefficient; *M* = mean, *SD* = standard deviation; *p* = *p* value; SWLS = satisfaction with life scale; PPS = perceived stress scale.

#### 3.1.4 High levels of value orientation associated with high levels of well-being (hypothesis 3)

Multiple mean comparisons using the Tukey's Honestly Significant Difference identified significant differences in the average levels of the well-being variables between *high*- and *low*-LVOs and between *moderate*- and *low*-LVOs (Table 5).

**Table 5:** Study 1: multiple pairwise comparisons using Tukey's honestly significant difference test.

	LVO	Diff.	CI	P
Comprehension	*moderate–low*	0.93	[0.578 – 1.284]	<.001
	*high–low*	1.21	[0.809 – 1.618]	<.001
	*high–moderate*	0.28	[-0.115 – 0.681]	.218
Purpose	*moderate–low*	1.03	[0.709 – 1.360]	<.001
	*high–low*	1.22	[0.851 – 1.597]	<.001
	*high–moderate*	0.19	[-0.178 – 0.556]	.449
Mattering	*moderate–low*	0.86	[0.467 – 1.252]	<.001
	*high–low*	1.17	[0.720 – 1.619]	<.001
	*high–moderate*	0.31	[-0.132 – 1.482]	.226
SWLS	*moderate–low*	1.12	[0.757 – 1.583]	<.001
	*high–low*	1.17	[0.753 – 1.910]	<.001
	*high–moderate*	0.05	[-0.360 – 0.457]	.958
PSS	*moderate–low*	-0.34	[-0.684 − 0.005]	<.001
	*high–low*	-0.45	[-0.843 − 0.065]	<.001
	*high–moderate*	-0.11	[-0.493 – 0.273]	.778

*Note*. *LVO* = level of value orientation; *Diff*. = mean difference; *CI* = confidence interval; *p* = *p* value; SWLS = satisfaction with life scale; PSS = perceived stress scale.

### 3.2 Study 2

Repeating the analysis conducted in study 1 aimed to validate the new measure of *values-as-ideals* and to replicate the results, which confirmed the three stated hypotheses.

#### 3.2.1 Values-as-ideals reliability

The *values-as-ideals* measure displayed very good internal reliability (α = .81).

#### 3.2.2 Three distinct levels of value orientation (hypothesis 1)

Conducting LPA on Sample 2 (Table 1) replicated the results of study 1. Similarly to the analysis in study 1, most of the fit indices of the 5-profile model did not converge (Table 6). Although *Entropy* indicated a 4-profile model as the best solution, the proportion of individuals assigned to the smallest group was less than 8% (*n.min* = .039), which made a 4-profile model unstable (Tein et al., 2013). Therefore, the 3-profile model was deemed to represent the data best (Nylund-Gibson and Choi, 2018; Tein et al., 2013).

**Table 6:** Study 2: fit statistics of latent profile analysis models as indicated by the values-as-ideals 10-item measure.

K	LL	BIC	SABIC	CAIC	AWE	Entropy	n_min
1	−6593	13490	13245	13329	13490	1	1
2	−6229	12649	12551	12680	12931	.78	.424
3	−6097	12452	12318	12494	12834	.82	.112
4	−6038	12402	12234	12455	12885	.86	.039
5	−5969	12331	12127	12395	12914	.85	.447

*Note*. *K* = number of profiles; *LL* = log-likelihood; *BIC* = bayesian information criterion; *SABIC* = sample size-adjusted BIC; *CAIC* = consistent Akaike information criterion; *AWE* = approximate weight of evidence; Entropy = a measure of classification uncertainty; *n_min* = proportion of the sample assigned to the smallest profile.

**Figure 3:**
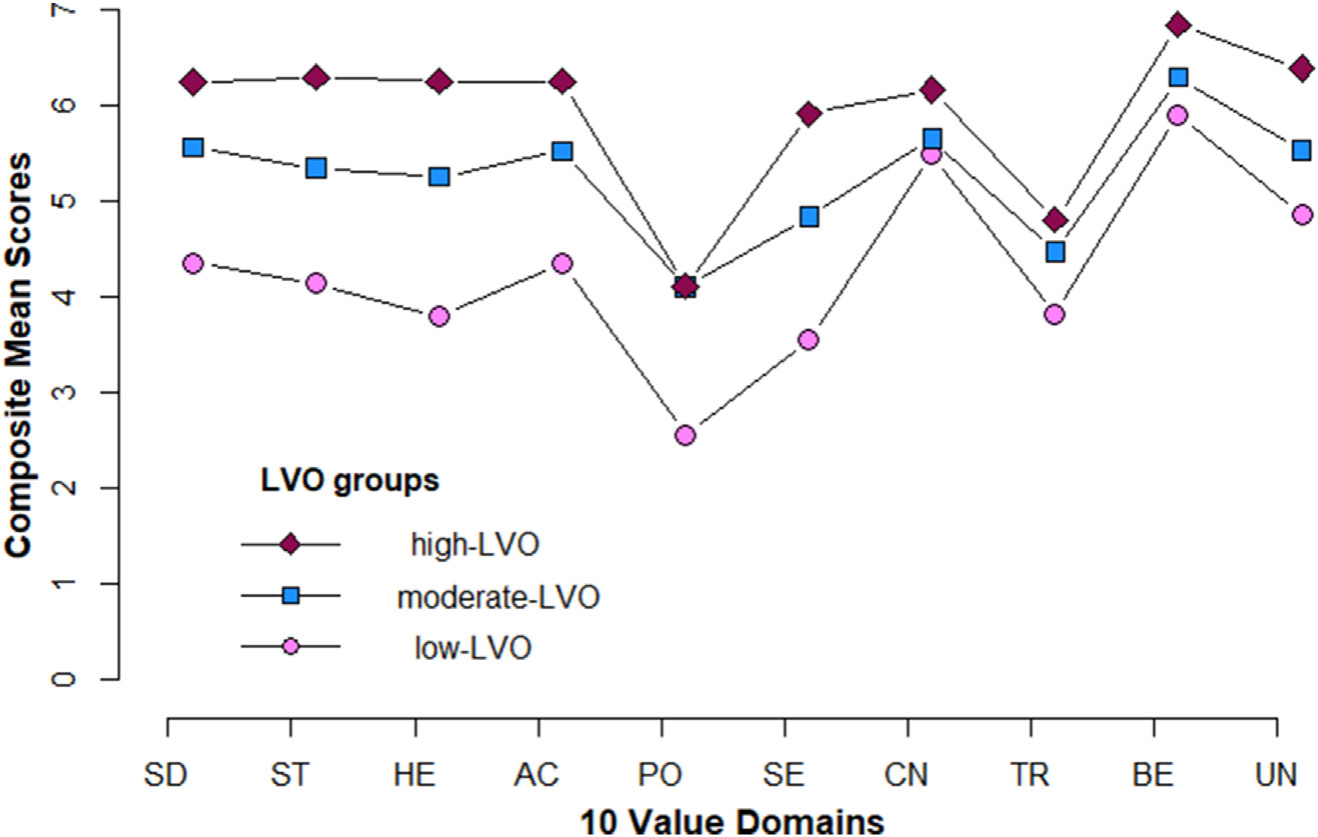
Sample 2 | Mean patterns of values across three groups distinguished by level of value orientation.

According to the item response probabilities by value ratings, 19.14% of participants (*n* = 96, age *M* = 43.64, *SD* = 13.95, females = 46) were estimated to belong to profile 1; 44.30% of participants (*n* = 215, age *M* = 42.51, *SD* = 13.40, females = 104) were estimated to belong to profile 2; and 36.56% of participants (*n* = 154, age *M* = 43.52, *SD* = 14.31, females = 83) were estimated to belong to profile 3. As in Sample 1, in profile1 the highest percentage of ratings of values on a 1 (*not at all*) to 7 (*very much*) point scale were gathered under points 4 and 5; in profile 2 the highest percentage of ratings was gathered under points 5 and 6, and in profile 3 the highest percentage of ratings was gathered under points 6 and 7 (Supplementary: Table 2). Hence, the interpretation of the three profiles as *low*-, *moderate*- and *high*-LVO (i.e., level of value orientation) were also meaningful in Sample 2. As in Sample 1, regardless of the level of value orientation they represented, the mean patterns of the three LVOs had a similar shape, with Power being the lower boundary and Benevolence being the upper boundary (Figure 3). In addition, the mean patterns of the three LVOs had a similar shape with the patterns of participants in Sample 1 (see Figures 2 and 3).

#### 3.2.3 Distinct levels of well-being across levels of value orientation (hypothesis 2)

In line with *Hypothesis 2*, ANOVA showed significant differences in average levels of the well-being measurements across LVOs. The well-being measurements were non-independent in LVO groups, which were distinguishable by their average levels of well-being (Table 7).

**Table 7:** Study 2: Well-being measurements were non independent across LVO groups.

Outcome	ICC(1)	ICC(2)	low-LVO		moderate-LVO		high-LVO		F(2, 468)	p
			M	SD	M	SD	M	SD		
Comprehension	.20	.97	3.35	1.34	4.59	1.16	4.95	1.16	40.63	<.001
Purpose	.23	.98	3.62	1.22	4.71	1.08	5.19	1.08	47.23	<.001
Mattering	.15	.96	2.87	1.43	4.01	1.33	4.92	1.33	27.53	<.001
SWLS	.15	.96	3.34	1.32	4.27	1.16	4.66	1.16	27.83	<.001
PSS	.08	.93	4.45	1.30	3.93	1.04	3.56	1.04	14.76	<.001

*Note. LVO* = level of value orientation; *ICC(1)* = interclass correlation coefficient; *ICC(2)* = intraclass correlation coefficient; *M* = mean, *SD* = standard deviation; *p* = *p* value; *SWLS* = satisfaction with life scale; PPS = perceived stress scale.

#### 3.2.4 High levels of value orientation associated with high levels of well-being (hypothesis. 3)

**Figure 4:**
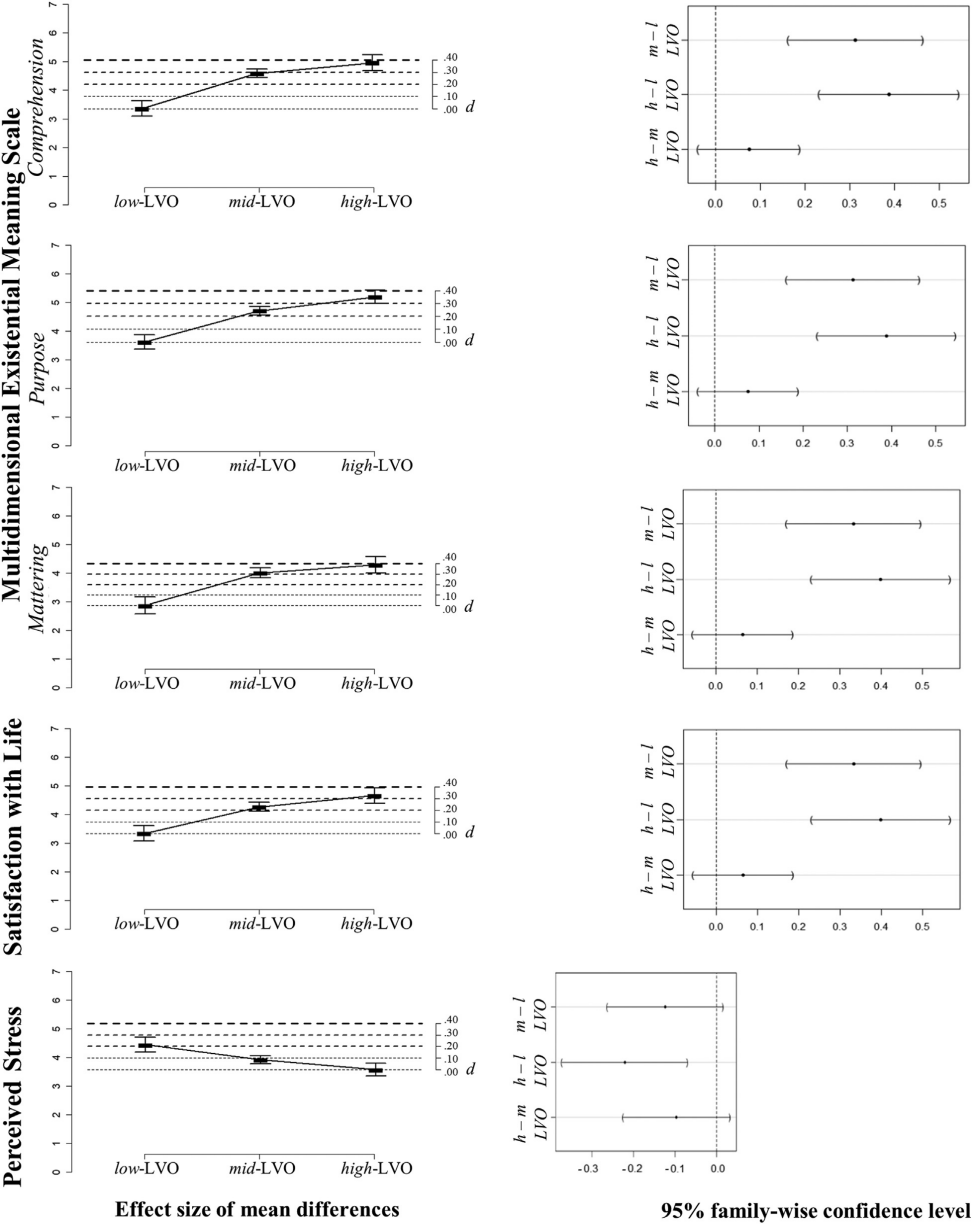
Multiple pairwise comparisons of average levels of well-being across three groups distinguished by levels of value orientation, showing effect size for differences between *low*, *moderate* and *high*-LVO and 95% confidence intervals.

Multiple pairwise comparisons between group means using the Tukey's Honestly Significant Difference test replicated the findings of study 1. In line with *Hypothesis 3*, average levels of well-being in the *high*-LVO group were significantly higher compared to the *low*-LVO (Figure 4 and Supplementary: Table 3).

#### 3.2.5 Validity

Replication of results across Sample 1 and Sample 2 supported the convergence and divergence validity of the new measure of *values-as-ideals*.

## 4 Discussion

Previous methodologies employed to investigate the relationship between values and well-being place an emphasis on people's value priorities and lead to a multiple conceptual dissection of the universal structure of values (e.g., conflicting vs complimentary, healthy vs unhealthy). Converging with the view that values do not have inherent characteristics *per se* (Schwartz, 1992), the present article considers a range of factors that possibly influence the relationship between values and well-being (e.g., value extremity, value congruence and a range of psychological factors) and may explain the randomly reported negative associations between certain values (e.g., Security) and well-being measurements (e.g., Subjective Well-Being) (e.g., Kasser and Ahuvia, 2002). The present work aims to shift the focus from the type of values people hold onto the level the universal values influence people as a dynamic pattern, by providing a new measure that assesses *values-as-ideals* and by proposing a new methodological approach. The present investigation of the relationship between value patterns and well-being yields results suggesting that the conceptual dichotomy of the circular structure of universal values into “conflicting” and “complimentary” or into “healthy” and “unhealthy” (e.g., Schwartz, 1992) constrains the understanding of the way the universal cognitive structure of values behaves and influences well-being. In line with studies indicating that single values do not predict well-being variables systematically (e.g., Oishi et al., 1999; Sortheix and Schwartz, 2017), this within-study cross-validation investigation provides empirical evidence suggesting that the level at which the universal values serve as people's ideals explains variation in average levels of well-being. Converging with previous work (Besika et al., 2022) participants with a high level of value orientation report higher degree of meaning and life satisfaction and lower degrees of stress than those with a low level of value orientation. These results are also in line with a recent study that used LPA to identify homogeneous clusters (Schmidt et al., 2021) based on people's ratings of the 21 items of PVQ (Schwartz, 2006). LPA identified three latent value profiles, which the authors interpret as negative, neutral or positive to values. However, this interpretation of the three profiles is not very meaningful without a theoretical background. Interpreting the three latent value profiles as different levels of value orientation converges with previous research suggesting that an increased capacity for integrating multiple life roles as parts of the self-concept increases ability to operate within multiple life domains, which is associated with high levels of self-esteem and low levels of depression (Marks and MacDermid, 1996). The present findings are also in line with previous work showing that the degree the 10 universal values influence a person's behavior and daily actions influences their ability to maintain overall well-being and adapt to change successfully (Besika et al., 2021).

It is worth highlighting that regardless of the level of value orientation, Power had commonly the lowest mean. Figure A in the Supplementary material illustrates that Power is commonly the lowest boundary in the mean pattern of young, middle-aged and old adults in a previous study that explored value differences across the life span using data from the World Value Survey (Freund and Ritter, 2014, p.11). Although it may be premature to draw any conclusions, the striking similarities in the shape of mean pattern of different samples may be meaningful and deserve further exploration. The finding that the value domain of Benevolence emerged as the highest rated ideal amongst all participants converges with many findings from the meaning literature. For example, studies that investigated the most common sources of meaning show that people derive meaning mostly from family and relationships (e.g., Baum and Stewart, 1990; O'Connor and Chamberlain, 1996). Further evidence suggests that close relationships (e.g., with a significant other), influence people's health and well-being significantly (Kiecolt-Glaser and Wilson, 2017; Seeman, 1996).

Previous research relies on value priorities to investigate variability in well-being. The new measure of *values-as-ideals* uses a direct way to assess the degree values serve as ideals that guide people's behavior. Unlike existing measures of values, the self-related statements included in the new measure aim to avoid activating self-discrepancies between actual and ideal self-states, as this may result in emotional discomfort (Higgins, 1987, 1997). Moreover, the 10 items assessing *values-as-ideals* displayed good reliabilities in contrast to existing measures (e.g.*,* Sortheix and Schwartz, 2017). The present work suggests that employing a new approach in future investigations may contribute to a better understanding of the way values influence well-being. Using latent value profiles as a unit of analysis may provide meaningful information regarding interindividual and intraindividual differences in well-being and other constructs.

## 5 Limitations

The sample of the present study poses some limitations in generalizing results as it is representative of the United Kingdom. Future research may administer the methodology of the present study on different cultures. In addition, the three emerging LVOs represent functioning individuals with low levels of stress. Future studies may investigate value orientation patterns of individuals who experience severe levels of stress and anxiety as a step toward identifying LVOs that are associated with dysfunction. Considering that individuals who participate in online research studies may have a low income, it is likely that the finding that the value of Power is the lowest boundary in the mean pattern of all three LVO groups reflects the participants' social status. Future studies may engage participants in power positions and investigate the shape of their value mean pattern in relation to their level of well-being. Finally, the present study is based on cross-sectional data that represent participants’ LVOs at one point in time. As the importance of values fluctuates over time, longitudinal studies may examine the temporal fluctuation of value patterns and the possible impact of such fluctuations on well-being.

## 6 Conclusion

In aiming to overcome the barriers raised in research progress by characterizing values as “conflicting” or “unhealthy”, the present article introduces a more reliable approach to investigating the value to well-being relationship. Findings indicate that all universal values may play an important role on the stage of life, as this comprises multiple domains. A replicated mean pattern across the three identified latent value profiles suggests that although a high level of value orientation may have a stronger association to well-being than a low level of value orientation, an optimal value pattern may not consist of high levels in all values. Similarly to a tango dancer who constantly balances different elements in their movement (e.g., leading and following, strength and softness, stillness and movement), an individual may develop the ability to orchestrate their personal values within a universal cognitive structure and manifest behaviors of unique characteristics. The present work may stimulate research interest in the underlying mechanism that contributes to the experience of well-being by translating values as universal meaning systems into personal goals and actions.

## Declarations

### Author contribution statement

Anastasia Besika: Conceived and designed the studies; Performed the studies; Analyzed and interpreted the data; Contributed reagents, materials, analysis tools or data; Wrote the paper.

### Funding statement

This work was supported by the ETH Dual Career Department.

### Data availability statement

Data will be made available on request.

### Declaration of interest’s statement

The author declares no conflict of interest.

### Additional information

Supplementary content related to this article has been published online at [URL].

## Appendix A Supplementary data

Supplementary_Revised Supplementary_Revised
