# Supplementary material for: A within-study cross-validation of the values-as-ideals measure: levels of value orientation explain variability in well-being
Source: Heliyon. 2022 Dec 9;8(12):e12131. doi: 10.1016/j.heliyon.2022.e12131 (PMC9792749; doi:10.1016/j.heliyon.2022.e12131)
Supplement: Supplementary_Revised [file mmc1.doc]

**Supplementary Material**

**Values-as-ideals Questionnaire**

- To what degree do the following statements describe you?

**In an ideal world:**

1. I decide about which way my life goes.

*Not at all* *1 2 3 4 5 6 7 Very much*

1. I discover new things in life.

*Not at all* *1 2 3 4 5 6 7 Very much*

1. I enjoy life to the fullest.

*Not at all* *1 2 3 4 5 6 7 Very much*

1. I am successful in everything I do.

*Not at all* *1 2 3 4 5 6 7 Very much*

1. I have resources and influence over others.

*Not at all* *1 2 3 4 5 6 7 Very much*

1. I feel safe wherever I am.

*Not at all* *1 2 3 4 5 6 7 Very much*

1. I respect other people and follow social rules*.*

*Not at all* *1 2 3 4 5 6 7 Very much*

1. I accept and follow the ideas of my culture or religion.

*Not at all* *1 2 3 4 5 6 7 Very much*

1. I care about my family, friends and others around me.

*Not at all* *1 2 3 4 5 6 7 Very much*

1. I care about all things on the planet.

*Not at all* *1 2 3 4 5 6 7 Very much*

| **Table 1** | | | | | | |  |
| --- | --- | --- | --- | --- | --- | --- | --- |
| *Study 1: Item-response probabilites in percentages (%) for a 3-profile model.* | | | | | | |  |
| *Profile 1* | | | | | | | |
|  | | | | | | | |
| *Rating* | *1* | *2* | *3* | *4* | *5* | *6* | *7* |
| *Self-Direction* | 01.19 | 02.86 | 08.36 | 24.21 | 33.13 | 27.42 | 02.83 |
| *Stimulation* | 00.61 | 03.59 | 07.77 | 26.62 | 44.96 | 14.70 | 01.76 |
| *Hedonism* | 01.79 | 04.77 | 13.81 | 31.05 | 39.80 | 07.67 | 01.12 |
| *Achievement* | 00.00 | 01.17 | 03.68 | 28.27 | 42.30 | 18.49 | 06.10 |
| *Power* | 13.25 | 19.42 | 22.72 | 31.71 | 12.90 | 00.00 | 00.00 |
| *Security* | 02.53 | 08.31 | 13.51 | 28.82 | 29.13 | 14.13 | 03.58 |
| *Conformity* | 00.00 | 00.90 | 02.39 | 11.58 | 32.55 | 36.73 | 15.86 |
| *Tradition* | 10.38 | 08.54 | 09.86 | 34.09 | 24.82 | 09.21 | 03.10 |
| *Benevolence* | 00.00 | 00.00 | 02.39 | 07.02 | 11.88 | 30.69 | 48.03 |
| *Universalism* | 01.22 | 01.79 | 07.92 | 17.47 | 25.71 | 28.85 | 17.05 |
| Total rating | 30.97 | 61.35 | 92.41 | **240.84** | **297.18** | 187.89 | 99.35 |
| *Profile 2* | | | | | | | |
| *Rating* | *1* | *2* | *3* | *4* | *5* | *6* | *7* |
| *Self-Direction* | 00.00 | 00.00 | 00.00 | 01.52 | 26.72 | 48.57 | 23.19 |
| *Stimulation* | 00.00 | 00.00 | 00.00 | 04.10 | 33.03 | 45.60 | 17.27 |
| *Hedonism* | 00.00 | 00.00 | 00.52 | 02.02 | 24.87 | 53.92 | 18.68 |
| *Achievement* | 00.00 | 00.00 | 00.99 | 04.08 | 25.77 | 51.72 | 17.44 |
| *Power* | 00.80 | 05.05 | 15.90 | 23.40 | 31.52 | 18.70 | 04.63 |
| *Security* | 00.00 | 00.00 | 04.40 | 10.88 | 33.33 | 30.51 | 20.89 |
| *Conformity* | 00.00 | 01.31 | 00.00 | 04.48 | 22.79 | 53.73 | 17.71 |
| *Tradition* | 06.83 | 04.39 | 04.35 | 24.71 | 20.87 | 30.61 | 08.24 |
| *Benevolence* | 00.00 | 00.00 | 00.00 | 01.17 | 11.54 | 31.51 | 55.77 |
| *Universalism* | 00.00 | 00.00 | 03.01 | 05.87 | 26.66 | 43.93 | 20.53 |
| Total | 07.63 | 10.54 | 28.18 | 82.23 | **257.10** | **380.80** | 205.06 |
| *Profile 3* | | | | | | | |
| *Rating* | *1* | *2* | *3* | *4* | *5* | *6* | *7* |
| *Self-Direction* | 01.82 | 02.91 | 00.00 | 05.04 | 04.24 | 09.67 | 76.32 |
| *Stimulation* | 02.71 | 01.81 | 00.89 | 06.90 | 16.20 | 15.98 | 55.51 |
| *Hedonism* | 03.62 | 00.92 | 00.80 | 08.29 | 12.72 | 13.24 | 60.40 |
| *Achievement* | 00.91 | 00.95 | 04.49 | 11.68 | 06.45 | 06.93 | 68.59 |
| *Power* | 24.75 | 06.21 | 06.98 | 23.93 | 16.71 | 05.83 | 15.60 |
| *Security* | 06.14 | 03.70 | 04.54 | 12.73 | 09.80 | 08.43 | 54.66 |
| *Conformity* | 03.63 | 01.81 | 00.91 | 11.86 | 02.83 | 14.78 | 64.18 |
| *Tradition* | 18.71 | 03.94 | 05.63 | 19.84 | 12.45 | 10.29 | 29.15 |
| *Benevolence* | 02.72 | 00.00 | 00.00 | 02.73 | 01.03 | 01.49 | 92.03 |
| *Universalism* | 02.68 | 00.00 | 00.00 | 05.97 | 07.46 | 10.97 | 72.91 |
| Total | 67.69 | 20.44 | 24.24 | 108.97 | 89.94 | **97.61** | **589.35** |
| *Note.* Text in bold indicates the total of the two ratings with the highest percentage in each profile | | | | | | | |

| **Table 2** | | | | | | |  |
| --- | --- | --- | --- | --- | --- | --- | --- |
| *Study 2: Item-response probabilites in percentages for a 3-profile model.* | | | | | | |  |
| *Profile 1* | | | | | | | |
|  | | | | | | | |
| *Rating* | *1* | *2* | *3* | *4* | *5* | *6* | *7* |
| *Self-Direction* | 02.06 | 03.92 | 09.83 | 42.88 | 23.25 | 10.73 | 07.33 |
| *Stimulation* | 00.00 | 06.86 | 17.62 | 36.33 | 29.26 | 07.86 | 02.07 |
| *Hedonism* | 02.94 | 10.78 | 20.71 | 35.17 | 24.50 | 04.42 | 01.48 |
| *Achievement* | 02.94 | 04.90 | 08.22 | 40.39 | 26.08 | 10.82 | 06.65 |
| *Power* | 29.28 | 21.98 | 18.71 | 20.93 | 07.62 | 00.00 | 00.00 |
| *Security* | 10.63 | 07.84 | 26.62 | 28.98 | 19.35 | 05.59 | 00.99 |
| *Conformity* | 00.00 | 00.00 | 03.51 | 19.20 | 28.05 | 26.18 | 23.06 |
| *Tradition* | 14.25 | 09.27 | 06.21 | 35.05 | 23.69 | 09.42 | 02.10 |
| *Benevolence* | 00.00 | 00.98 | 00.00 | 13.92 | 19.76 | 22.57 | 42.77 |
| *Universalism* | 00.00 | 00.00 | 06.74 | 29.80 | 36.44 | 19.70 | 07.31 |
| Total rating | 62.10 | 66.53 | 118.17 | **302.65** | **238.00** | 119.29 | 53.55 |
| *Profile 2* | | | | | | | |
| *Rating* | *1* | *2* | *3* | *4* | *5* | *6* | *7* |
| *Self-Direction* | 00.00 | 00.00 | 00.00 | 08.48 | 37.86 | 43.07 | 10.59 |
| *Stimulation* | 00.00 | 00.00 | 00.00 | 13.01 | 41.00 | 41.48 | 04.51 |
| *Hedonism* | 00.00 | 00.00 | 00.00 | 14.00 | 47.94 | 34.32 | 03.75 |
| *Achievement* | 00.00 | 00.00 | 00.29 | 07.12 | 40.05 | 43.05 | 09.49 |
| *Power* | 02.10 | 08.04 | 18.68 | 32.79 | 25.78 | 11.34 | 01.26 |
| *Security* | 00.00 | 02.83 | 12.49 | 19.02 | 32.18 | 28.03 | 05.46 |
| *Conformity* | 00.00 | 00.00 | 01.14 | 06.18 | 29.40 | 49.75 | 13.58 |
| *Tradition* | 06.44 | 03.66 | 10.17 | 21.98 | 31.34 | 23.35 | 03.05 |
| *Benevolence* | 00.00 | 00.47 | 00.47 | 00.85 | 14.07 | 34.06 | 50.08 |
| *Universalism* | 00.00 | 00.47 | 01.42 | 10.08 | 34.81 | 37.96 | 15.26 |
| Total | 08.54 | 15.47 | 44.57 | 133.59 | **334.43** | **346.41** | 107.03 |
| *Profile 3* | | | | | | | |
| *Rating* | *1* | *2* | *3* | *4* | *5* | *6* | *7* |
| *Self-Direction* | 01.26 | 00.00 | 01.96 | 02.15 | 12.58 | 27.65 | 54.40 |
| *Stimulation* | 01.99 | 00.00 | 00.67 | 02.21 | 13.38 | 23.86 | 57.89 |
| *Hedonism* | 01.99 | 00.00 | 02.56 | 02.26 | 12.82 | 22.34 | 58.03 |
| *Achievement* | 01.32 | 00.66 | 00.00 | 07.08 | 09.58 | 23.63 | 57.73 |
| *Power* | 14.36 | 04.98 | 10.13 | 30.56 | 17.60 | 08.90 | 13.47 |
| *Security* | 00.76 | 00.00 | 05.53 | 10.67 | 14.59 | 21.12 | 47.34 |
| *Conformity* | 01.99 | 01.99 | 01.32 | 07.48 | 06.64 | 21.72 | 58.86 |
| *Tradition* | 09.80 | 03.83 | 05.36 | 21.62 | 18.80 | 17.70 | 22.79 |
| *Benevolence* | 00.66 | 00.00 | 00.00 | 01.98 | 00.00 | 07.12 | 90.24 |
| *Universalism* | 01.99 | 00.66 | 00.73 | 02.36 | 01.46 | 24.12 | 63.10 |
| *Total* | 36.12 | 12.12 | 28.26 | 301.22 | 59.09 | **198.16** | **523.85** |
| *Note.* Text in bold indicates the total of the two ratings with the highest percentage in each profile | | | | | | | |

| **Table 3** | | | | | | |
| --- | --- | --- | --- | --- | --- | --- |
| *Study 2: Multiple pairwise comparisons using Tukey’s Honestly Significant Difference test.* | | | | | | |
| Outcome | *LVO* | *Diff.* | *CI* | | | *p* |
| Comprehension | *moderate - low* | 1.23 | [0.829 | – | 1.635] | <.001 |
|  | *high - low* | 1.60 | [1.173 | – | 2.027] | <.001 |
|  | *high - moderate* | 0.37 | [0.023 | – | 0.715] | .034 |
| Purpose | *moderate - low* | 1.09 | [0.726 | – | 1.447] | <.001 |
|  | *high - low* | 1.57 | [1.188 | – | 1.952] | <.001 |
|  | *high - moderate* | 0.48 | [0.173 | – | 0.793] | .008 |
| Mattering | *moderate - low* | 1.14 | [0.701 | – | 1.577] | <.001 |
|  | *high - low* | 1.42 | [0.953 | – | 1.881] | <.001 |
|  | *high - moderate* | 0.28 | [-0.099 | – | 0.655] | .192 |
| SWLS | *moderate - low* | 0.93 | [0.535 | – | 1.326] | <.001 |
|  | *high - low* | 1.32 | [0.899 | – | 1.737] | <.001 |
|  | *high - moderate* | 0.39 | [0.388 | – | 0.048] | .021 |
| PPS | *moderate - low* | -0.52 | [-0.873 | – | -0.162] | .002 |
|  | *high - low* | -0.87 | [-1.247 | – | -0.493] | <.001 |
|  | *high - moderate* | -0.35 | [-0.658 | – | -0.046] | .019 |
| Note. *LVO* = level of value orientation; *Diff*. = mean difference; *CI* = confidence interval;  *p* = *p* value; SWLS = satisfaction with life scale; PSS = perceived stress scale. | | | | | | |


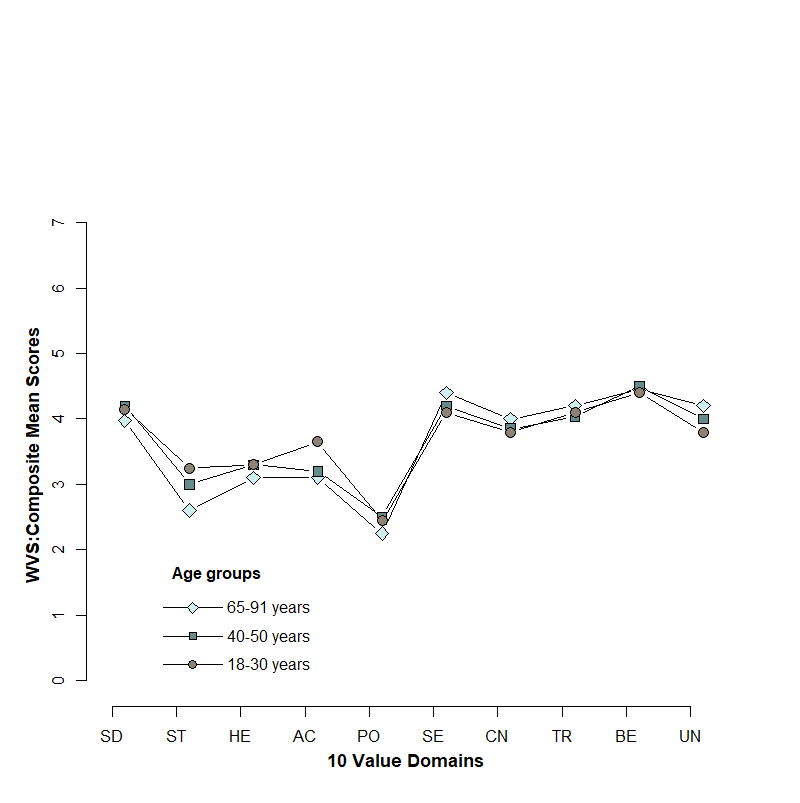


**Figure A:** Mean patterns of values across 3 age groups, based on Figure 18.2 in Freund & Ritter (2014, p.15). (SD = Self-direction, ST = Stimulation, HE = Hedonism, AC = Achievement, PO = Power, SE = Security, CN = Conformity, TR = Tradition, BE = Benevolence, UN = Universalism).
